# Resistance exercise promotes the resolution and recanalization of deep venous thrombosis in a mouse model via SIRT1 upregulation

**DOI:** 10.1186/s12872-022-02908-y

**Published:** 2023-01-13

**Authors:** Caijiao Wu, Xiaorong Li, Huihan Zhao, Ying Ling, Yanping Ying, Yu He, Shaohan Zhang, Shijing Liang, Jiani Wei, Xiao Gan

**Affiliations:** 1 Department of Nursing, The First Affiliated Hospital of Guangxi Medical University, Nanning, 530021 Guangxi China; 2 Department of Critical Care Medicine, The First Affiliated Hospital of Guangxi Medical University, Nanning, Guangxi China; 3 Medical Lab, The First Affiliated Hospital of Guangxi Medical University, Nanning, 530021 Guangxi China

**Keywords:** Resistance exercise, Deep venous thrombosis, Resolution, Sirtuin 1

## Abstract

**Background:**

Early exercise for acute deep venous thrombosis (DVT) improves the patient’s symptoms and does not increase the risk of pulmonary embolism. However, information about its effect on thrombus resolution is limited. The aim of this study was to investigate the role of resistance exercise (RE) in thrombus resolution and recanalization and determine its underlying mechanisms.

**Methods:**

Ninety-six C57BL/6 J mice were randomly divided into four groups: Control group (C, *n* = 24); DVT group (D, *n* = 24); RE + DVT group (ED, *n* = 24); and inhibitor + RE + DVT group (IED, *n* = 24). A DVT model was induced by stenosis of the inferior vena cava (IVC). After undergoing IVC ultrasound within 24 h post-operation to confirm DVT formation, mice without thrombosis were excluded. Other mice were sacrificed and specimens were obtained 14 or 28 days after operation. Thrombus-containing IVC was weighed, and the thrombus area and recanalization rate were calculated using HE staining. Masson’s trichrome staining was used to analyze the collagen content. RT-PCR and ELISA were performed to examine IL-6, TNF-α, IL-10, and VEGF expression levels. SIRT1 expression was assessed using immunohistochemistry staining and RT-PCR. VEGF-A protein expression and CD-31-positive microvascular density (MVD) in the thrombus were observed using immunohistochemistry.

**Results:**

RE did not increase the incidence of pulmonary embolism. It reduced the weight and size of the thrombus and the collagen content. Conversely, it increased the recanalization rate. It also decreased the levels of the pro-inflammatory factors IL-6 and TNF-α and increased the expression levels of the anti-inflammatory factor IL-10. RE enhanced VEGF and SIRT1 expression levels and increased the MVD in the thrombosis area. After EX527 (SIRT1 inhibitor) was applied, the positive effects of exercise were suppressed.

**Conclusions:**

RE can inhibit inflammatory responses, reduce collagen deposition, and increase angiogenesis in DVT mice, thereby promoting thrombus resolution and recanalization. Its underlying mechanism may be associated with the upregulation of SIRT1 expression.

## Background

Deep venous thrombosis (DVT) is a common vascular disease with an annual incidence of 0.1% in the general population and > 1% in hospitalized individuals [1]. The acute phase of DVT develops into pulmonary embolism, which threatens patients’ lives. Late-stage DVT can lead to post-thrombosis syndrome (PTS), which seriously affects the quality of life of patients [2]. Current therapies for DVT rely on anticoagulation, thrombolysis, and compression [3, 4]. Anticoagulation prevents subsequent DVT extension but does not significantly accelerate thrombus resolution and reduce the occurrence of PTS [5]. Thrombolytic therapy can reduce residual thrombosis and restore partial blood flow but can only be used in a certain subset of patients and is associated with considerable bleeding risk [6]. Compression therapy can relieve pain and edema, but a multicenter RCT study found that compression cannot reduce the incidence of PTS and recurrent venous thromboembolism [7]. In addition, the latest guidelines do not advocate for the routine use of compression in proximal DVT [8, 9]. Thus, safe, economical, and effective strategies must be developed to accelerate thrombolysis and recanalization for DVT and reduce the occurrence of PTS. Thrombus resolution is similar to granulation tissue formation during wound healing [10]. This process involves angiogenesis, fibrinolysis, and inflammatory responses [11, 12]. Exercise can promote fibrinolysis, regulate inflammatory responses, and promote angiogenesis in myocardial infarction [13], cerebral infarction [14, 15], atherosclerosis [16, 17], thrombosis [18]. It also relieves the symptoms of DVT, prevents the further extension of thrombi, and reduces the risk of PTS [19–22]. The potential mechanisms of exercise are closely related to thrombus resolution and recanalization but have not been directly reported.

Sirtuins are highly conserved NAD^+^-dependent deacetylases, and seven subtypes have been identified [23]. Among all sirtuins, SIRT1 (silent information regulator 2 homolog 1) is expressed in all organization types, especially in the vasculature [24, 25]. It targets a wide range of transcription factors and participates in many physiological and pathological processes, such as oxidative stress, inflammation, angiogenesis, and energy metabolism [26–29]. Upregulation of SIRT1 can reduce the production of pro-inflammatory factors and decrease the thrombosis burden. Conversely, EX527, a SIRT1 inhibitor, increases the protein expression of Ace-p65, which leads to an increased expression of downstream pro-inflammatory factors, thereby exacerbating the burden of thrombosis [30, 31]. Recent studies have demonstrated that SIRT1 regulates angiogenic activity and promotes angiogenesis [29]. Resveratrol, a SIRT1 agonist, promotes angiogenesis and accelerates the recanalization of DVT [32]. Studies found that inhibition of SIRT1 impairs angiogenesis through downregulation of vascular endothelial growth factor A (VEGF-A) [33, 34]. VEGF is a potent angiogenic cytokine. When thrombi are treated with recombinant VEGF protein or plasmid containing VEGF, thrombus recanalization and resolution are accelerated [35, 36]. In addition, *in-vivo* and *in-vitro* studies found that overexpression of SIRT1 can enhance migration and proliferation of endothelial progenitor cells (EPCs) and promote angiogenesis [37, 38]. Therefore, SIRT1 may promote angiogenesis by upregulating VEGF and suppressing inflammation, which plays an important role in thrombus resolution and recanalization. Furthermore, the regulation of SIRT1 expression may be a potential target for DVT therapy.

Exercise can significantly upregulate SIRT1 activation and expression, regulate inflammation, and increase angiogenesis [39–42]. For example, El et al. [43] found that exercise can upregulate SIRT1 expression and promote angiogenesis in ischemic areas. However, whether exercise can upregulate SIRT1 expression in deep vein thrombosis has not been reported. On this basis, we aimed to investigate whether resistance exercise (RE) accelerates thrombus resolution and recanalization through the upregulation of SIRT1 expression. Our results provide new evidence for exercise to treat deep vein thrombosis.

## Methods

### Animal grouping

Ninety-six C57/BL6J mice (male, weight 23–25 g, 9 weeks of age) were purchased from Beijing Sibafu Biotechnology Co. (Experimental Animal Breeding License Number SCXK [Jing] 2019–0010). They were housed in a climate-controlled room (20–25 °C, 50–60% humidity, and 12 h/12 h light/dark cycles) with food and water ad libitum. Before the operation, they were subjected to an adaptive RE schedule (two sets/day, three times/set) over a 3-day acclimation period and randomly divided into four groups according to a table of random numbers: Control group (C, *n* = 24); DVT group (D, *n* = 24); RE + DVT group (ED, *n* = 24); and inhibitor + RE + DVT group (IED, *n* = 24). The mice in each group were equally and randomly divided into 14- and 28-day groups. All procedures were approved by the Animal Care and Welfare Committee of Guangxi Medical University (approval number: 202103031). This protocol faithfully complied with the Guidelines for Laboratory Animal Care and Use of the National Institutes of Health (NIH Publication No. 85–23). This study was conducted in accordance with the ARRIVE guidelines. All animals were euthanized using Lethal dose 2% isoflurane at the end of the experiment. Utmost efforts were made to reduce the number of animals used and minimize their suffering.

### DVT induced by the stenosis of the inferior vena cava (IVC) in mice

A DVT model was established by stenosis of the IVC [44–47]. Briefly, the mice were anesthetized via the inhalation of 2% isoflurane with 100% oxygen and then routinely disinfected. Next, a 2.0-cm incision was made along the midline laparotomy, and subcutaneous tissues in the abdominal cavity were separated to expose the IVC. The IVC was ligated just below the left renal vein and IVC border triangle area using a 4–0 silk suture. A 30-G spacer was placed parallel to the IVC, and a 4–0 silk suture was used to partially ligate the IVC to 10% of its original diameter. The spacer was removed, and the mouse was closed in a layered fashion. Intra- and post-operative mice were kept at 37 °C with a heating pad, and normal saline was injected subcutaneously (0.2 ml/10 g) after surgery. The mice were returned to their cages with free access to food and water 2 h post-operation. The control group did not receive treatment or surgery for baseline comparison.

### High-frequency ultrasound method

Ultrasound is the recommended approach for thrombosis detection because of its high sensitivity, specificity, and noninvasive characteristics [48]. In the present study, the mice were subjected to IVC ultrasound (M7, Mindray, Shenzhen, China) within 24 h of surgery to determine thrombus formation. First, they were anesthetized via isoflurane gas inhalation (2%) with a mixture of 100% oxygen. Then, they were depilated on the abdomen and placed in a supine position. Their temperature was monitored using a rectal probe. The IVC was visualized using an ultrasound probe and examined in axial views from the renal veins to the iliac bifurcation to observe IVC, stenosis, and thrombus formation. An optimal freeze-frame image was manually obtained. Pulse-wave Doppler imaging and color Doppler imaging were used to quantify blood flow velocity [47].

### Experimental intervention

**Fig. 1:**
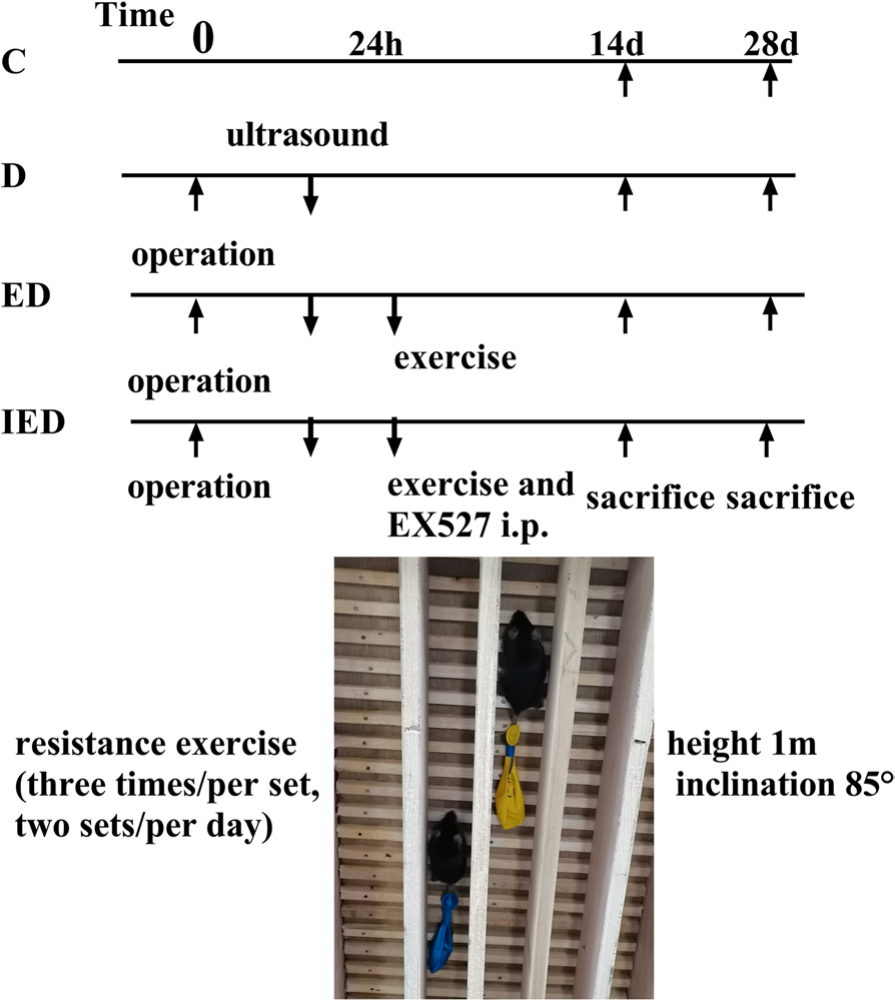
Experimental design and apparatus used in this study. C Control group, D DVT group, ED exercise + DVT, IED EX527 + exercise + DVT (SIRT1 inhibitor) group. Mice in the ED and IED groups were forced to climb the ladder (three times/set, two sets/day, 6 days/week for 14 and 28 days)

Ultrasound was used to confirm thrombosis, and the mice without thrombosis were removed. After DVT was confirmed via ultrasound, the mice in the exercise (ED and IED) groups were allowed to use a 1-m ladder composed of a 1-cm grid inclined at 85° [49]. As they climbed the ladder until they reached the top, appropriate weights were attached to their tails. They were trained 6 days/week and rested for 1 day. Two sets were trained three times daily per group for 2 min. Training was conducted for 4 weeks. The loaded weights were equivalent to 10, 30, 50, and 70% of their body weight at weeks 1, 2, 3, and 4, respectively. The IED group was subjected to exercise and administered EX527 (HY15452, MedChemExpress, USA), a selective SIRT1 inhibitor (10 mg/kg, 6 days/week, i.p.). EX527 was dissolved in dimethyl sulfoxide (DMSO) and diluted in normal saline to a final DMSO concentration of < 2% [30]. EX527 was administered to the mice 30 min before RE. The experimental design is illustrated in Fig. 1.

### Serum preparation and tissue harvest

The mice were anesthetized with 2% isoflurane, and blood samples were collected and centrifuged (3000 × *g*, 4 °C) for 15 min for supernatant collection, which was stored at − 80 °C for further use. The mice were euthanized using Lethal dose 2% isoflurane, and the IVC with its associated thrombus was harvested and weighed as thrombus weight [46, 50], which is a means of evaluating thrombus resolution. Subsequently, parts of the thrombosed IVC were fixed in 10% paraformaldehyde, embedded in paraffin, and sliced into 4-μm sections for hematoxylin and eosin (HE) staining, Masson’s trichrome staining, and immunohistochemistry staining. Other parts of the thrombosed IVC were fixed by snap-freezing in liquid nitrogen and stored at − 80 °C for RT-PCR analysis. Lung tissues were randomly selected for HE staining at 14 and 28 days in each group to determine pulmonary embolism.

### Enzyme-linked immunosorbent assays

ELISA kits, namely, ml063159 (mlbio, Shanghai, China), mI002095 (mlbio), ml037873 (mlbio), and KJ-2864B (Kejing, Jiangsu, China), were used to quantify interleukin-6 (IL-6), tumor necrosis factor-alpha (TNF-α), interleukin-10 (IL-10), and vascular endothelial growth factor (VEGF) levels, respectively, in accordance with the manufacturer’s instructions. Optical density was examined at 450 nm using a microplate reader (Varioskan LUX, Thermo Fisher), and the IL-6/TNF-α/IL-10/VEGF content was calculated using a standard curve.

### Hematoxylin and eosin staining

IVC and lung tissue slices were dewaxed with xylene (I) for 5 min and toluene (II) for 5 min, dehydrated using graded ethanol, and washed with distilled water for 2 min. Then, they were stained with hematoxylin for 5 min, differentiated with 1% hydrochloric acid ethanol for 30 s, immersed in tap water for 15 min, placed in eosin solution for 2 min, and slightly washed with distilled water for 2 min. Afterward, slices were dehydrated, cleared, and fixed with neutral resin. They were observed under a microscope (BX53F, Olympus, Japan). The vein recanalization rate (% area of lumen) and thrombus area (% area of vein lumen) were analyzed using Image-Pro Plus version 5.0 [51]. The pathological sections of the lungs were examined to determine the occurrence of pulmonary embolism. Each image was analyzed by two independent researchers.

### Masson’s trichrome analysis for thrombosis (solid green method)

Deparaffinized sections were stained with Weiger iron–hematoxylin for 10 min, differentiated with 1% hydrochloric acid ethanol for 3 s, washed with water, and allowed to turn blue for 2 min. Afterward, they were stained with ponceau-acid fuchsin solution for 5 min and rinsed with 3% glacial acetic acid for 1 min. Sections were further stained with 1% phosphomolybdic acid for 3 s and treated with 3% glacial acetic acid for 1 min. They were directly counterstained with Solid green staining solution (G1661, Solarbio) for 5 min and sealed with neutral resin. Collagen fibers were dyed green. Images were taken with an Olympus BX53F microscope, and the percentage of the collagen content in the thrombus was quantified through computerized analysis using Image-Pro Plus version 5.0 [52].

### Immunohistochemical analyses

Deparaffinized sections were subjected to immunohistochemistry as previously described. Antigens were retrieved on the slides using 10 mM sodium citrate and heated in a pressure cooker for 10 min. The slides were then immersed in H_2_O_2_ in methanol for 10 min to eliminate endogenous peroxidase activity. The sections were incubated with Goat serum for 10 min to reduce nonspecific reactions. They were then incubated with anti-CD31 pAbs (GB11063-2, Servicebio, Wuhan, China); antiSIRT1mAbs (ab110304, Abcam, USA), and anti-VEGFA mAbs (ab52917, Abcam) at 4 °C overnight. The slides were incubated with goat resistant mouse IgG polymer for 10 min. Then, they were incubated with HRP for 10 min. Color development was performed using a DAB substrate for approximately 5 min, and the specimens were counterstained with hematoxylin. The sections were examined under a microscope. SIRT1 and VEGF-A protein expression levels were quantified by blindly calculating the average optical density (AOD) in Image-Pro Plus (version 5.0). Each image was analyzed to obtain the integrated optical density (IOD) and area of the pixel (AREA) of a tissue. AOD was determined using the following equation: (AOD = IOD/AREA) [53]. Intrathrombotic CD31-positive microvascular density (MVD) was measured [34]. All measurements were performed by an examiner without prior knowledge of the experimental procedure.

### Real-time quantitative polymerase chain reaction

Total RNA was extracted from frozen samples using an RNA extraction kit (Invitrogen, USA). The purity of the isolated RNA was determined by measuring absorbance at 260/280 nm using a spectrophotometer (Nanodrop One, Thermo Scientific). Next, RNA was reverse-transcribed into cDNA using the HyperScript III RT Super Mix Kit (EnzyArtisan, China). Real-time quantitative polymerase chain reaction (RT-qPCR) was performed using 2X S6 Universal SYBR qPCR Mix on an ABI 7500 instrument (Applied Biosystems, USA). β-Actin was used as an internal control for other genes. Fold changes were calculated using the 2-^ΔΔCt^ method. All primers were synthesized by EnzyArtisan Co., Ltd. (Shanghai, China; Table 1).

**Table 1:** Primer sequences for RT-qPCR

Genes	Primer sequences	Expected length (bp)
SIRT1	F: 5’-CGGCTACCGAGGTCCATATAC-3’	109
	R: 5’-CAGCTCAGGTGGAGGAATTGT-3’	
VEGF	F: 5’-GCAGACTATTCAGCGGACTCA-3’	146
	R: 5’-CCGTTGGCACGATTTAAGAGG-3’	
IL-6	F: 5’-ACAAAGCCAGAGTCCTTCAGAG-3’	75
	R: 5’-TGTGACTCCAGCTTATCTCTTGG-3’	
IL-10	F: 5’-TGCAGTGTGTATTGAGTCTGCT-3’	119
	R: 5’-TCGGAGAGAGGTACAAACGAG-3’	
β-Actin	F: 5’-CATTGCTGACAGGATGCAGAAGG-3’	138
	R: 5’-TGCTGGAAGGTGGACAGTGAGG-3’	

*F* Forward primer, *R* Reverse prime

### Statistical analysis

Quantitative data are presented as mean ± standard deviation (SD). They were analyzed using SPSS 22.0 for Windows (SPSS Inc., Chicago, IL, USA) and GraphPad Prism 8.1.2. Differences among multiple groups were analyzed using one-way analysis of variance (ANOVA) followed by Bonferroni’s test for homogeneity of variance or Tamhane’s test for unequal variances. Statistical significance was set at *P* < 0.05.

## Results

### Visualizing venous thrombosis through ultrasonography

**Fig. 2:**
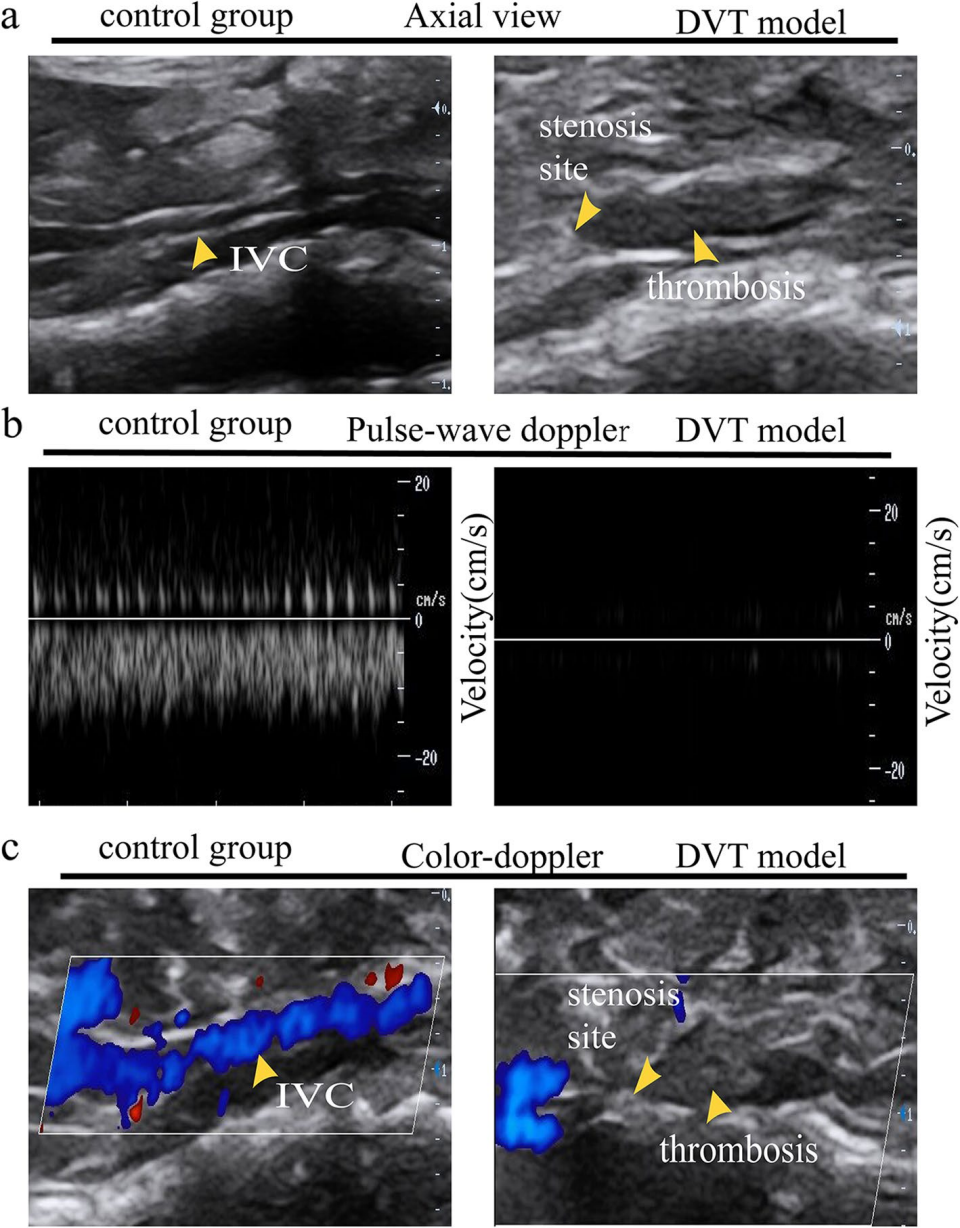
Monitoring of the thrombus formation of the inferior vena cava (IVC) through ultrasound imaging. **a** Representative images of the IVC in the axial view in the control group (left panel) and DVT model within 24 h (right panel). A thrombus is visualized in axial views. **b** Representative images of blood flow velocity depicted by using pulse-wave Doppler; no flow was observed in the DVT mouse model. **c** Blood flow velocity can also be depicted by color Doppler. In the DVT mouse model, the absence of flow is depicted by the absence of color

All mice were examined using ultrasound. In the control group, two-dimensional axial ultrasound could visualize the vascular wall of the vein. Pulse-wave Doppler imaging revealed normal blood flow, and color Doppler showed normal blood flow. The criteria for the successful establishment of the DVT mouse model included the following: two-dimensional axial ultrasound showed hypoechogenic thrombi in the vessels distal to the stenosis site; pulse-wave Doppler showed no blood flow; and color Doppler detection showed no blood flow in the absence of color in the DVT mouse model (Fig. 2). Thrombosis was observed in 62 mice: 10 in D14, 11 in D28, 10 in ED14, 11 in ED28, 10 in IED14, and 10 in IED28. In this study, the rate of thrombosis was 86.1%.

### RE did not increase pulmonary embolism

**Fig. 3:**
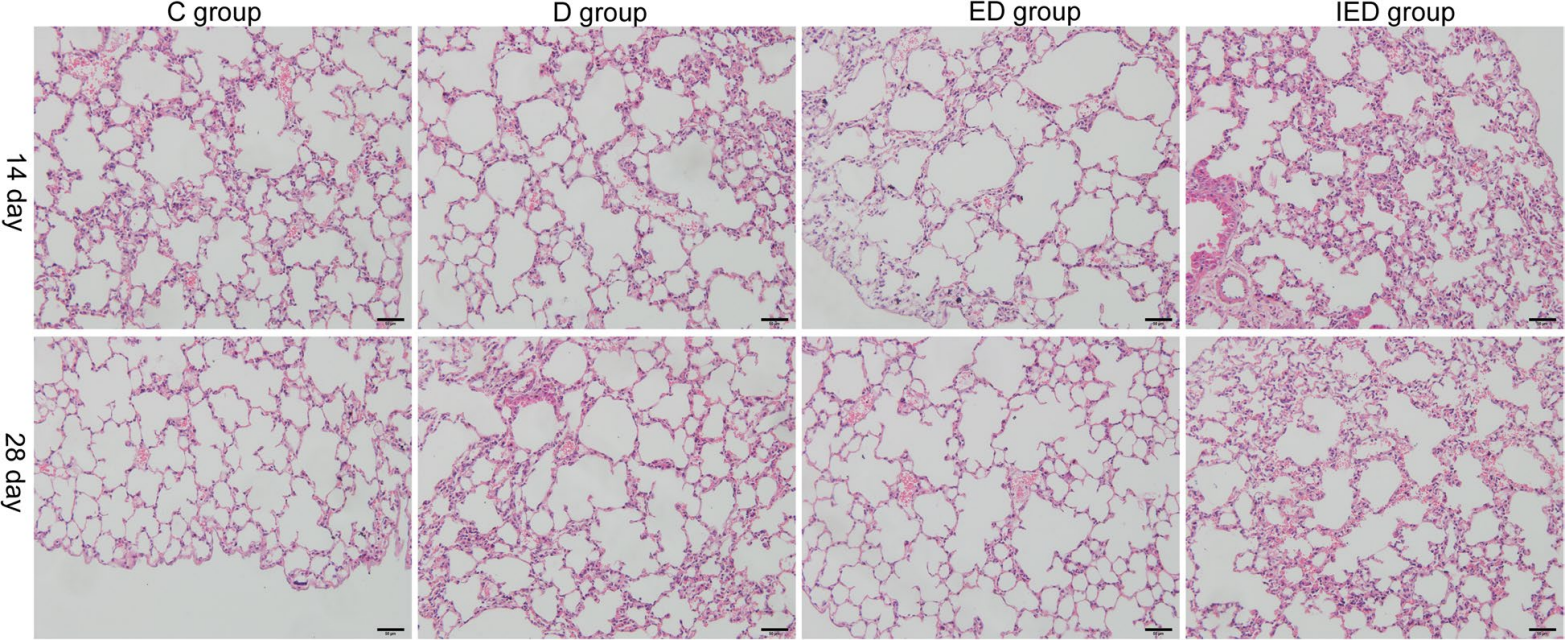
Typical HE-stained sections of lung in mice in C, D, ED, or IED group. Magnification × 400. Scale bar: 50 µm. No pulmonary embolism was observed in all groups (*n* = 5 mice). C: Control group; D: DVT group; ED: exercise and DVT group; IED: SIRT1 inhibitor, exercise DVT group

To investigate whether exercise is safe in mice with deep vein thrombosis, we analyzed the lung tissues using HE staining. HE staining showed no pulmonary embolism of each group (Fig. 3).

### EX527 reversed the RE-induced decrease in the weight and size of the thrombus and increase in the vein recanalization rate

**Fig. 4:**
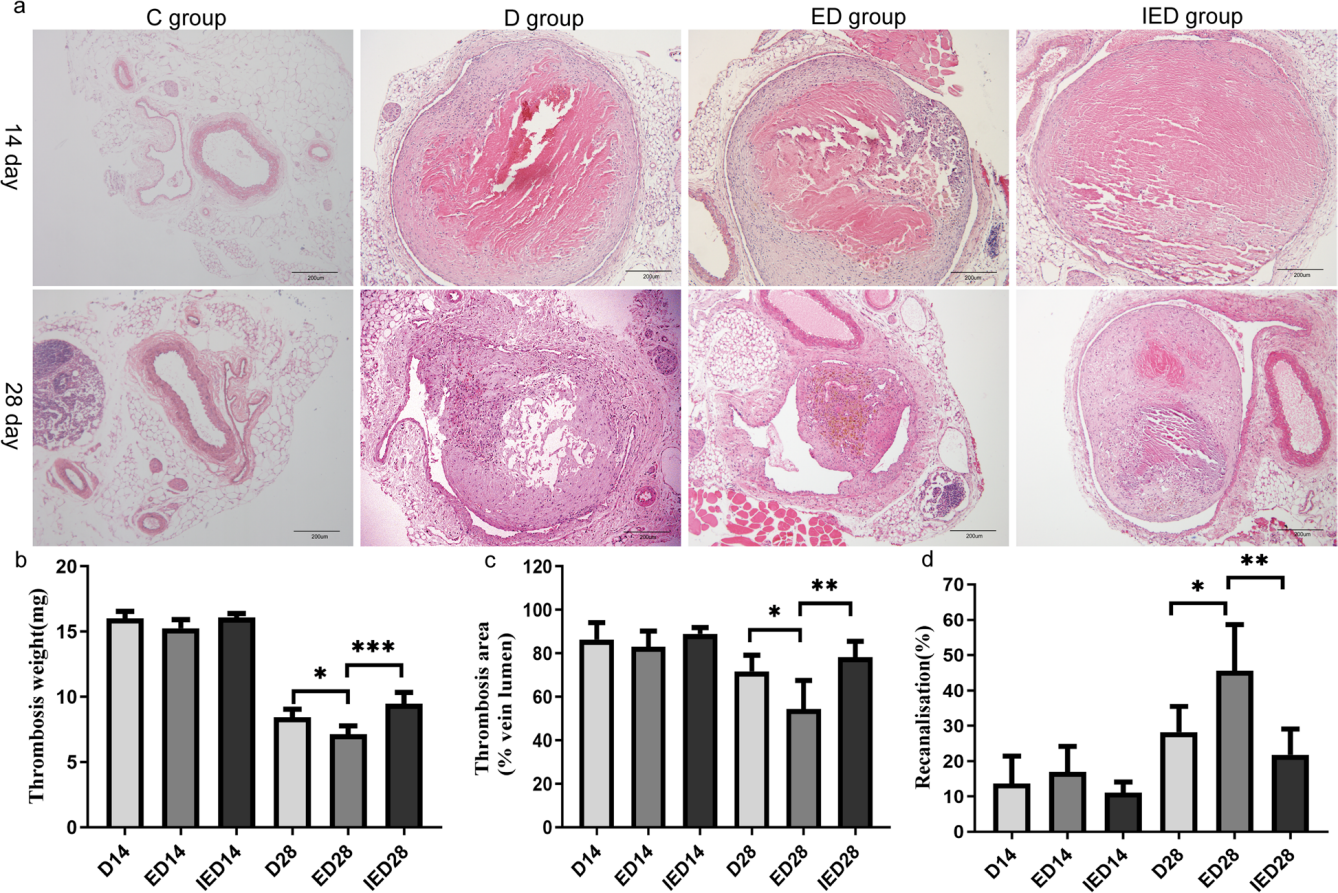
Thrombus weight, size, and vein recanalization rate on days 14 and 28. (**a**) Original magnification, × 100. Representative thrombus sections stained with hematoxylin and eosin in C, D, ED, and IED groups. Scale bar: 200 μm. Thrombus weight (**b**), thrombus size (**c**), and vein recanalization rate (**d**) are shown. Thrombus size is expressed as a percentage of the total vein lumen area, while recanalization rate is calculated as a percentage of the area of the vein lumen. All values represent the mean ± SD (*n* = 4–5 mice). Data were analyzed using ANOVA (*P < 0.05, **P < 0.01, ***P < 0.001).C: Control group; D: DVT group; ED: exercise and DVT group; IED: SIRT1 inhibitor, exercise DVT group

We assessed thrombus resolution and recanalization in mice by comparing their thrombus weight, thrombus size, and vein recanalization rate. The groups had no significant differences on day 14 (*P* > 0.05). RE significantly reduced thrombus weight and size in the ED group on day 28 compared with those in the D group (*P* < 0.05). The vein recanalization rate of the mice in the ED group was significantly higher than that of the mice in the D group on day 28 (*P* < 0.05). However, thrombus weight and size significantly increased, and recanalization rate significantly decreased in the IED group on day 28 compared with those in the ED group (*P* < 0.05, Fig. 4).

### RE reduced collagen deposition within the venous thrombi and affected inflammatory factor levels, but EX527 reversed these results

**Fig. 5:**
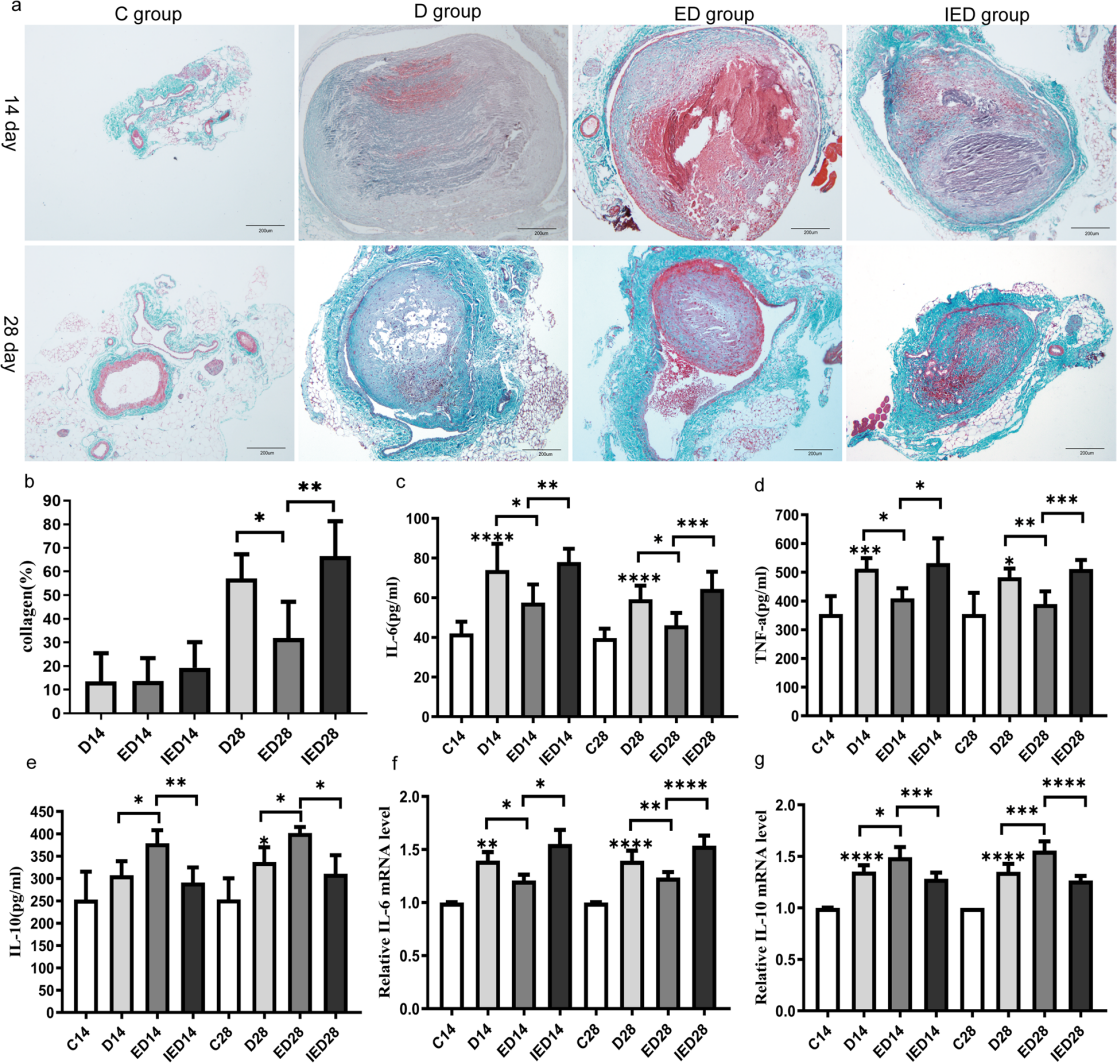
Collagen content and IL-10, IL-6, and TNF-a expression levels on days 14 and 28. (**a**) Masson’s trichrome staining of the IVC of the groups at the indicated time points; representative pictures are shown. Magnification: × 100. Scale bar: 200 µm. (**b**) Summary of the quantitative analysis of the collagen contents of venous thrombi for MTC staining per group on days 14 and 28 (*n* = 3–5 mice). IL-6 (**c**), TNF-a (**d**), and IL-10 (**e**) protein concentrations of the mice in each group were measured through ELISA on days 14 and 28 (*n* = 6 mice). mRNA expression levels of IL-6 (**f**) and IL-10 (**g**) within the thrombus were determined through RT-qPCR (*n* = 5 mice). Data are expressed as the means ± SD and analyzed through one-way ANOVA (*P < 0.05, **P < 0.01, ***P < 0.001, ****P < 0.0001). C: Control group; D: DVT group; ED: exercise and DVT group; IED: SIRT1 inhibitor, exercise DVT group

To study the effect of RE on thrombus collagen deposition, we determined the intrathrombotic collagen content using Masson’s trichrome staining. The D, ED, and IED groups did not differ on day 14 (*P* > 0.05). However, on day 28, the collagen content of the thrombus in the ED group was significantly lower than that in the D group (*P* < 0.05). The collagen content of the mice with SIRT1 inhibition was higher in the IED group than in the ED group (*P* < 0.05). We used ELISA and PCR to analyze the protein and mRNA expression levels of IL-6, TNF-a, and IL10. The expression levels of IL-6, TNF-α, and IL-10 were significantly higher in the D group on days 14 and 28 than in the control group (*P* < 0.05). The mRNA and protein expression levels of IL-10 were significantly higher in the ED group than in the D group, whereas those of IL-6 and TNF-α were lower, on days 14 and 28 (*P* < 0.05). The mRNA and protein expression levels of IL-6 and TNF-α were significantly higher in the IED group than in the ED group, whereas those of IL-10 were significantly lower in the IED as compared to the ED group on days 14 and 28 (*P* < 0.05, Fig. 5).

### RE increased CD31-positive MVD, but EX527 reversed this result

**Fig. 6:**
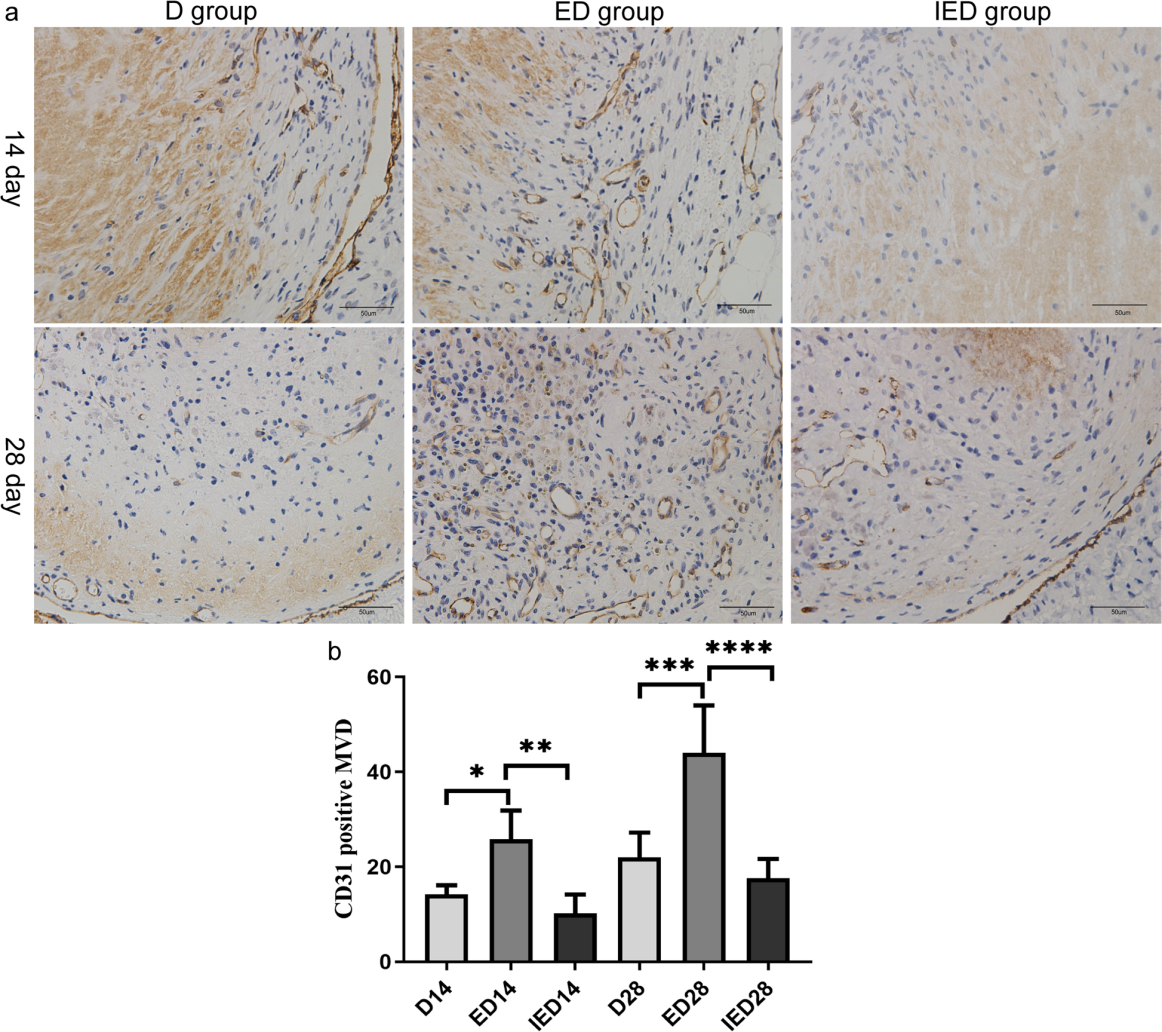
CD31-positive MVD on days 14 and 28. **a** Immunohistochemical staining of the CD31-positive MVD of thrombosis in each group of mice on days 14 and 28, and typical images are shown. Scale bar: 50 µm. **b** Quantitative analysis of CD31-positive MVD in D, ED, and IED groups on days 14 and 28. Data are represented as means ± SD (*n* = 5 mice) and were analyzed through one-way ANOVA (*P < 0.05, **P < 0.01, ***P < 0.001, ****P < 0.0001). D: DVT group; ED: exercise and DVT group; IED: SIRT1 inhibitor, exercise DVT group

MVD was significantly higher on days 14 and 28 (*P* < 0.05) in the ED group than in the D group. MVD was significantly suppressed in the IED group compared with that in the ED group on days 14 and 28 (*P* < 0.05, Fig. 6).

### RE increased VEGF expression and EX527 suppressed RE-induced VEGF expression

**Fig. 7:**
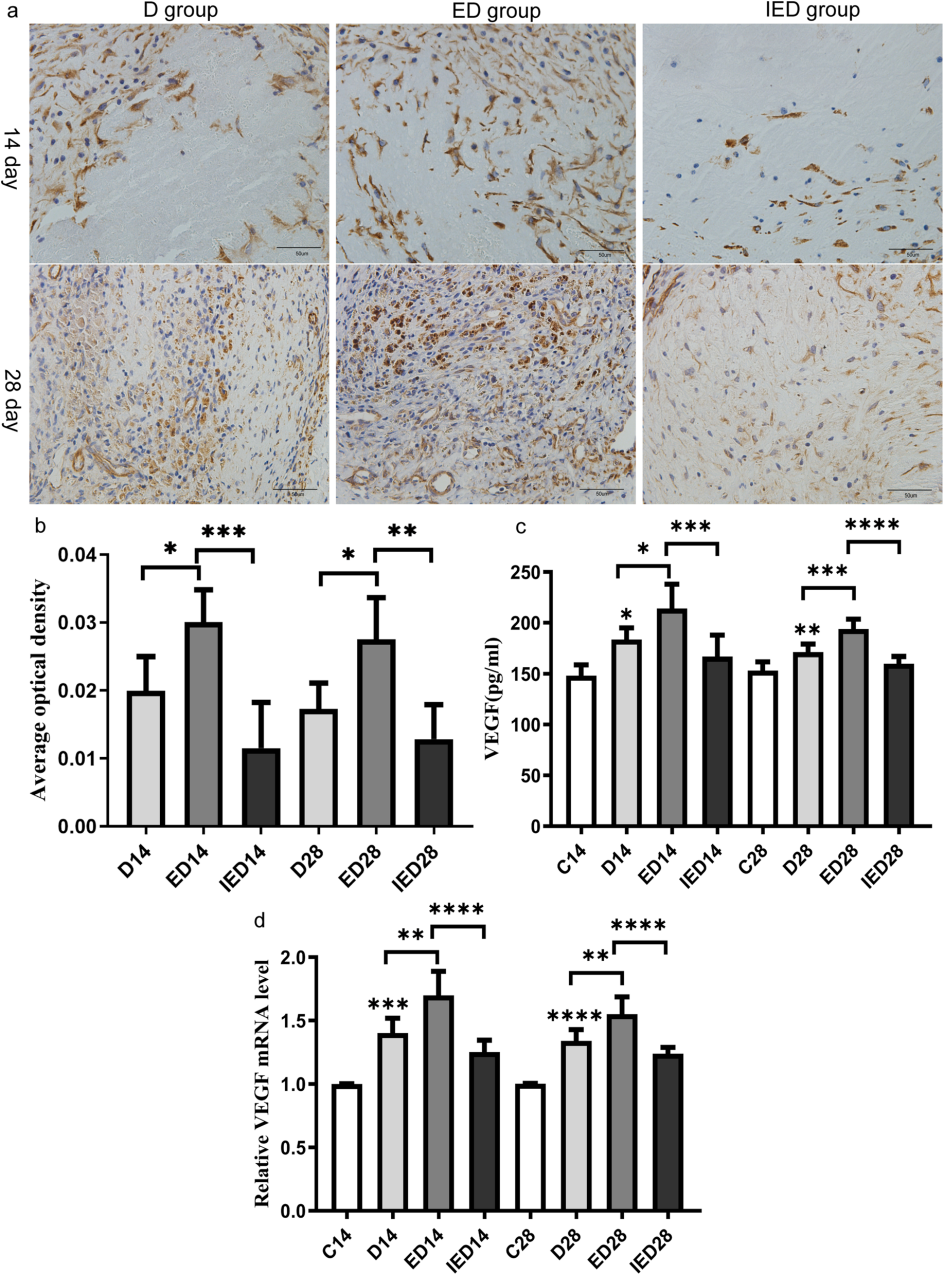
Expression of VEGF on days 14 and 28. **a** Immunohistochemical (IHC) staining images of VEGFA of the different groups on days 14 and 28. Scale bar: 50 µm. **b** Quantitative analysis and comparison of the AOD of IHC staining of VEGFA in each group of mice on days 14 and 28 (*n* = 5 mice). ELISA (**c**) results of the VEGF protein expression in serum (*n* = 6 mice). **d** Analysis of the relative mRNA expression of VEGF in the thrombosed IVC via RT-PCR (*n* = 5 mice). Data are expressed as means ± SD and were analyzed through one-way ANOVA (*P < 0.05, **P < 0.01, ***P < 0.001, ****P < 0.0001). C: Control group; D: DVT group; ED: exercise and DVT group; IED: SIRT1 inhibitor, exercise DVT group

We detected VEGF-A protein using immunohistochemistry. The AOD of VEGF-A was significantly higher in the ED group than in the D group on days 14 and 28 (*P* < 0.05). SIRT1 inhibition reduced the AOD of VEGF-A in the IED group compared with that in the ED group (*P* < 0.05). Serum ELISA and RT-q PCR results showed that VEGF protein and mRNA levels were significantly higher in the D group than in the control group on days 14 and 28 (*P* < 0.05). The protein and mRNA expression levels of VEGF were significantly higher in the ED group than in the D group at the 14 and 28 days (*P* < 0.05). Furthermore, SIRT1 inhibition was associated with decreased VEGF protein and mRNA expression in the IED group compared with that in the ED group on days 14 and 28 (*P* < 0.05, Fig. 7).

### RE increased SIRT1 expression, but EX527 suppressed RE-induced SIRT1 expression

**Fig. 8:**
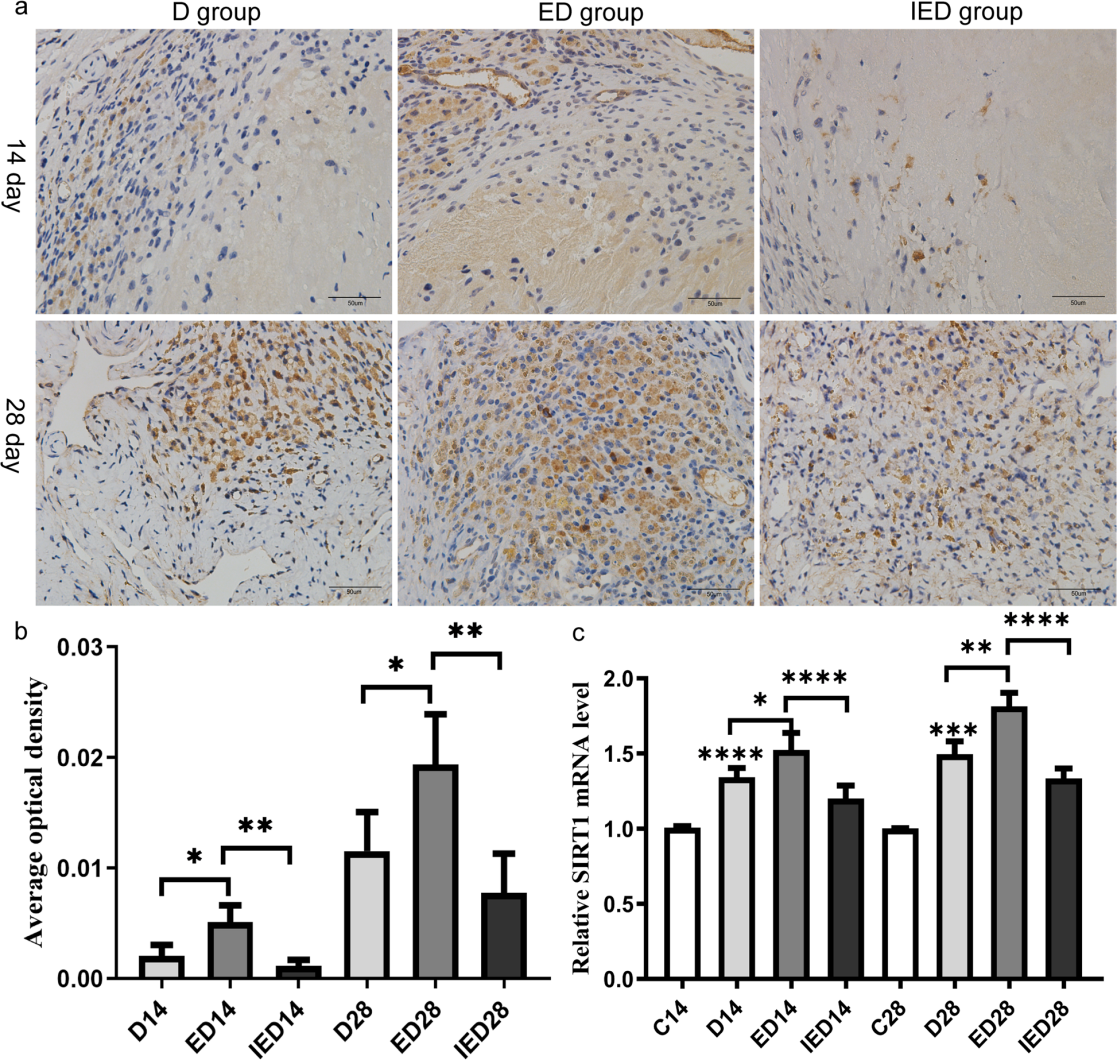
**a** Expression of SIRT1 on days 14 and 28. Immunofluorescence staining of SIRT1 among the groups on days 14 and 28, and representative images are shown here. Scale bar: 50 µm. **b** Comparison of the AOD of IHC staining of SIRT1 in each group of mice on days 14 and 28 (*n* = 4–5 mice). **c** RT-PCR analysis of the relative mRNA expression of SIRT1 in the thrombosed IVC (*n* = 5 mice). Data are expressed as means ± SD and were analyzed through ANOVA (*P < 0.05, **P < 0.01, ***P < 0.001, ****P < 0.0001). C: Control group; D: DVT group; ED: exercise and DVT group; IED: SIRT1 inhibitor, exercise DVT group

Immunohistochemical results showed that the AOD values of SIRT1 in the ED group were higher than those in the D group on days 14 and 28 (*P* < 0.05). The AOD levels in the IED group were higher than those in the ED group (*P* < 0.05). RT-PCR results showed that the mRNA expression of SIRT1 was significantly higher in the D group than in the control group on days 14 and 28 (*P* < 0.05). The mRNA expression levels of SIRT1 were significantly higher in the ED group than in the D group at days 14 and 28 (*P* < 0.05). The mRNA expression of SIRT1 was significantly lower in the IED group than in the ED group on days 14 and 28 (*P* < 0.05, Fig. 8).

## Discussion

In the present study, we examined the effects of RE on thrombus resolution and recanalization in mice with DVT. We found that exercise reduced thrombus weight and size and increased the recanalization rate. Exercise-induced improvements in these effects were associated with the upregulation of SIRT1 expression. To verify our hypothesis, we used EX527 to block the effect of SIRT1 and found beneficial effects of exercise on thrombus resolution. This result suggested that the exercise-induced improvement in DVT mice was associated with SIRT1 activation.

DVT is a relatively common and potentially life-threatening condition [54]. Despite remarkable improvements in the prevention and treatment of DVT in recent years, approximately 30% of patients with DVT develop pulmonary embolism, and 20%–50% of patients develop PTS, which can increase the economic burden of patients, seriously affect their quality of life, and even threaten their life and health [2, 55]. Thrombolysis is a spontaneous, time-consuming process. First, thrombosis contracts and retracts from the vein wall, leading to the appearance of pockets and fissures around the thrombus, and inflammatory cells infiltrate the periphery of the thrombus. These new vascular channels may coalesce and restore the blood flow through the occluded vein. At the late stage of thrombolysis, vascular channels and inflammatory cells also appear within the body of the thrombus; some channels traverse the thrombus and connect to the vessel lumen. After 3 or 4 weeks, organization is well-advanced, and part of the vein lumen is recanalized [12, 56]. Angiogenesis, fibrinolysis, and inflammatory responses play important roles in this process [11]. The collagen content of the thrombus also increases over time, and more collagen deposition further hinders thrombus resolution, which is closely related to PTS [57, 58]. Patients with PTS develop clinical symptoms such as leg pain and swelling. Thus, an increase in neovascularization, reduction of inflammation, and decrease in collagen content likely promote thrombus resolution and reduce PTS occurrence.

Exercise has been used as an effective tool to prevent DVT [59]. It increases venous blood flow to reduce venous stasis and hypercoagulability [60]. Venous stasis, endothelial injury, and hypercoagulability are factors contributing to thrombosis [61]. Bed rest was previously considered a part of clinical treatment to prevent thrombotic propagation and reduce the risk of pulmonary embolism [20]; however, this strategy has been seriously questioned. Many recent clinical studies have reported that exercise is sufficiently safe for DVT because it does not increase the incidence of pulmonary embolism [19, 62–64]. Systematic review also supports this point of view [22]. In the present study, we found no signs of pulmonary embolism in the lung pathology of the mice in the ED group, suggesting that RE was safe and did not increase the occurrence of pulmonary embolism. In addition, exercise helps reduce symptoms, increase muscle strength, and improve walking function [22, 65, 66]. These results indicate that exercise training rather than traditional instructions to avoid exercise should be part of the treatment program for DVT. In our study, we found that the weight and size of the thrombus were lower in the RE group than in the D group, and the vein recanalization rate increased. Therefore, exercise can promote thrombus resolution and recanalization.

That exercise affects DVT may involve a variety of factors. In physically active individuals and patients with peripheral arterial disease, exercise training enhances endogenous fibrinolytic activity, manifested as a decrease in plasminogen activator inhibitor and an increase in tissue-type (tPA) and urokinase-type plasminogen activator (uPA) activity; these results suggest that regular exercise improves fibrinolytic activity [67–69]. Clinical and animal studies have shown that exercise training reduces inflammation while increasing the levels of IL-10 and reducing the levels of pro-inflammatory cytokines such as IL-6, IL-8, and TNF-α [16, 70]. Inflammation is one of the key mechanisms of thrombolysis and recanalization [71]. Decreased IL-6 expression inhibits collagen deposition and promotes thrombus resolution [72–74]. IL-10 injection reduces thrombus weight [56, 75]. Increased IL-10 levels negatively regulate collagen content and reduce fibrosis [76]. In the present study, the mice that underwent RE showed reduced serum IL-6 and TNF-α levels and increased serum IL-10 levels. The gene expression levels of IL-6 and TNF-α in the thrombus also increased, whereas the mRNA expression of IL-10 decreased after RE. In addition, the collagen content of the thrombus was reduced in the RE group. Our data also showed that RE could modulate inflammatory responses against DVT.

Exercise can also increase neovascularization [42, 77]. Histopathological analysis of different stages from thrombosis to thrombolysis has shown that the thrombolytic recirculation area has a high degree of neovascularization [12, 78]. The high density of angiogenesis in the thrombolysis area is closely related to fast thrombus resolution, enhanced prognosis, and low mortality in patients with DVT [78–80]. VEGF is the most important and effective vascular stimulator that plays an important role in neovascularization [81, 82]. VEGF upregulation promotes angiogenesis and accelerates thrombolysis and recanalization [36, 51, 83]. The underlying mechanisms may act locally by stimulating the growth of vessels from the vessel wall into the thrombus (angiogenesis) and upregulate tPA and uPA activities in endothelial cells, thereby enhancing local fibrinolysis [84]. Additionally, VEGF may mobilize systemic endothelial progenitor cells and increase endothelial cell survival [85, 86]. It has been proven that exercise upregulates the protein expression of VEGF and increases angiogenesis in cardiovascular and cerebrovascular diseases [87–91]. In our study, VEGF expression levels were significantly higher in the ED group than in the D group; the MVD was higher in the ED group than in the D group, suggesting that RE increased VEGF expression and promoted angiogenesis in mice with DVT.

SIRT1, an NAD^+^-dependent deacetylase, deacetylates histones and various non-histone substrates, such as p53, NF-κB, FOXOs, PGC1α, and PARP, which are involved in different pathophysiological processes, such as restriction, oxidative stress, inflammation, angiogenesis, and apoptosis [92]. SIRT1 is a key regulator of angiogenesis and vascular growth. It upregulates the expression of VEGF and promotes angiogenesis in cardiovascular diseases [24, 28, 33, 34, 93, 94]. FOXO1 is an important negative regulator of angiogenesis, and SIRT1 induces VEGF by deacetylating FOXO1 [28, 95]. SIRT1 also promotes EPC migration, differentiation, and proliferation; EPCs are essential for angiogenesis [33, 96]. The intravenous injection of EPCs treated with Resveratrol (a SIRT1 agonist) promotes thrombus resolution in a murine model of venous thrombosis in vivo. Further mechanism study found that RSV promotes angiogenesis [32]. In endothelial cells, SIRT1 silencing is accompanied by impaired angiogenic function in vitro [28, 38, 97, 98]. SIRT1 is also well-known for its antioxidant and anti-inflammatory properties. Upregulation SIRT1 decreases the levels of inflammatory factors, such as TNF-α and IL-6, further reducing thrombosis. Inhibiting SIRT1 expression can increase inflammatory factor levels [30, 31, 99–101]. SIRT1 regulates inflammation by deacetylating histone H3, NF-κB, HIF1a, and AP-1 [27].

SIRT1 is regulated by several phenolic plant extracts, vitamins, and caloric restrictions [102, 103]. One of its natural activators is exercise [104]. Exercise significantly upregulates SIRT1 expression and protects endothelial cell function. Further studies have shown that EX527 markedly reduces the therapeutic effects of exercise training [39, 105]. Exercise promotes SIRT1 expression possibly through the following mechanism. Exercise can activate AMPK, increase intracellular NAD^+^ levels, and induces SIRT1 expression by increasing shear stress [106–108]. It also increases lactic acid levels and indirectly activates SIRT1 [43]. In the present study, the protein and RNA expression levels of SIRT1 in the RE group were significantly higher than those in the D group. SIRT1 inhibition by EX527 increases the weight and size of the thrombus and reduces the recanalization rate. MVD, VEGF, and IL-10 expression levels decreased in the IED group, whereas collagen content, IL-6, and TNF-α expression increased. Our results suggested that RE promoted thrombus resolution and recanalization by upregulating SIRT1 expression.

To the best of our knowledge, this study provides the first evidence that RE significantly affects venous thrombus resolution without increasing pulmonary embolism. These results provide evidence for clinicians and patients regarding the use of adjunctive exercise therapy. However, this study had several limitations. First, the IVC stenosis model in animals is different from clinical DVT, possibly limiting the applicability of the conclusions for clinical practice. Second, we were unable to use Doppler ultrasound to dynamically observe changes in the thrombus. Third, this study did not include conventional clinical treatment, such as anticoagulation, which is inconsistent with the clinical patient treatment guidelines. Current guidelines on venous thrombosis have few detailed exercise recommendations, but exercise is theoretically beneficial to cardiovascular disease. Thus, the effect of RE on deep vein thrombosis and related mechanisms should be further studied to provide a theoretical basis for clinical practice.

## Conclusions

In this study, we elucidated the potential mechanisms of RE-mediated venous thrombus resolution and recanalization in DVT. We first observed that RE promoted thrombus resolution by activating angiogenesis and inhibiting inflammation in the thrombus in the DVT model. We also found that SIRT1 levels were upregulated in the thrombus after RE administration. Moreover, EX527, a specific inhibitor of SIRT1, inhibited angiogenesis and increased RE-induced inflammation. These results suggest that RE promotes thrombolysis and recanalization by promoting SIRT1 expression and provide a theoretical basis for applying RE as an adjunctive therapy for DVT.

## Data Availability

The datasets used and/or analyzed during the current study are available from the corresponding author on reasonable request.

## Abbreviations

DVT: Deep venous thrombosis
IVC: Inferior vena cava
ELISA: Enzyme-linked immunosorbent assay
RT-PCR: Real-time quantitative polymerase chain reaction
MVD: Microvascular density
PTS: Post-thrombosis syndrome
RCT: Randomized controlled trial
SIRT1: Silent information regulator 2 homolog 1
VEGF-A: Vascular endothelial growth factor A
DMSO: Dimethyl sulfoxide
HE: Hematoxylin and eosin
IL-6: Interleukin-6
TNF-α: Tumor necrosis factor-alpha
IL-10: Interleukin-10
VEGF: Vascular endothelial growth factor
EPC: Endothelial progenitor cell
AOD: Average optical density
IOD: Integrated optical density
tPA: Tissue-type plasminogen activator
uPA: Urokinase-type plasminogen activator
EPC: Endothelial progenitor cell
RSV: Resveratrol
